# Predictive value of clinical features and CT radiomics in the efficacy of hip preservation surgery with fibula allograft

**DOI:** 10.1186/s13018-023-04431-y

**Published:** 2023-12-08

**Authors:** Peng Xue, Hongzhong Xi, Hao Chen, Shuai He, Xin Liu, Bin Du

**Affiliations:** 1 The First School of Clinical Medicine of Nanjing University of Chinese Medicine, Nanjing, 210029 China; 2 Department of Orthopedics, The Affiliated Hospital of Nanjing University of Chinese Medicine, Hanzhong Road 155, Nanjing, 210029 China

**Keywords:** Osteonecrosis of femoral head, Fibula allograft, Hip preservation surgery, Prediction model, Radiomics

## Abstract

**Background:**

Despite being an effective treatment for osteonecrosis of the femoral head (ONFH), hip preservation surgery with fibula allograft (HPS&FA) still experiences numerous failures. Developing a prediction model based on clinical and radiomics predictors holds promise for addressing this issue.

**Methods:**

This study included 112 ONFH patients who underwent HPS&FA and were randomly divided into training and validation cohorts. Clinical data were collected, and clinically significant predictors were identified using univariate and multivariate analyses to develop a clinical prediction model (CPM). Simultaneously, the least absolute shrinkage and selection operator method was employed to select optimal radiomics features from preoperative hip computed tomography images, forming a radiomics prediction model (RPM). Furthermore, to enhance prediction accuracy, a clinical-radiomics prediction model (CRPM) was constructed by integrating all predictors. The predictive performance of the models was evaluated using receiver operating characteristic curve (ROC), area under the curve (AUC), DeLong test, calibration curve, and decision curve analysis.

**Results:**

Age, Japanese Investigation Committee classification, postoperative use of glucocorticoids or alcohol, and non-weightbearing time were identified as clinical predictors. The AUC of the ROC curve for the CPM was 0.847 in the training cohort and 0.762 in the validation cohort. After incorporating radiomics features, the CRPM showed improved AUC values of 0.875 in the training cohort and 0.918 in the validation cohort. Decision curves demonstrated that the CRPM yielded greater medical benefit across most risk thresholds.

**Conclusion:**

The CRPM serves as an efficient prediction model for assessing HPS&FA efficacy and holds potential as a personalized perioperative intervention tool to enhance HPS&FA success rates.

## Introduction

A refractory orthopedic disorder known as ONFH is thought to cause considerable hip joint dysfunction and potentially disability [1]. The femoral head lesion will get worse as the situation worsens, eventually leading to the femoral head collapsing [2]. It cannot be repaired after that and needs to be replaced with an artificial joint. Implementing efficient and timely intervention strategies are therefore essential to treating ONFH [3]. Currently, almost all surgeons concur that the patient's own joints should be kept as much as possible intact [4, 5]. According to the literature, HPS&FA had a significant success rate for hip preservation before artificial joint replacement [6–8]. However, HPS&FA still faces the following two obstacles. (1) Due to the lack of exact evaluation tools for preoperative patient screening, some ineligible patients will nonetheless have hip preservation failure. (2) There are no accurate methods for predicting the outcome of patients following HPS&FA.

Predictive models can diagnose and prognosticate [9]. Currently, it is used to differentiate diseases, screen cases, and predict efficacy [10, 11]. Several clinical predictors obtained from blood analysis and follow-up data have been assessed to predict the probability of femoral head collapse [12, 13]. Hip CT is critical for diagnosis of ONFH. Historically, just a few CT imaging features could be subjectively evaluated by physicians. Size, position, and cumulative range of ONFH were difficult to quantify. Radiomics provides a new option for maximizing imaging data [14]. Quantitative imaging features that reflect region heterogeneity can be extracted from radiomics. Predicting HPS&FA success may require the development of a predictive model and the screening of meaningful clinical predictors. However, there are no studies on preoperative patient selection and risk prediction models for the failure of hip preservation in HPS&FA.

To address the aforementioned difficulties, we developed a CRPM that combines radiomics features and clinical predictors and evaluated its performance internally. Our objectives are to: (1) identify suitable candidates for HPS&FA, (2) predict postoperative failure risks, and (3) offer relevant perioperative intervention guidance.

## Materials and methods

### Patient selection

**Fig.1:**
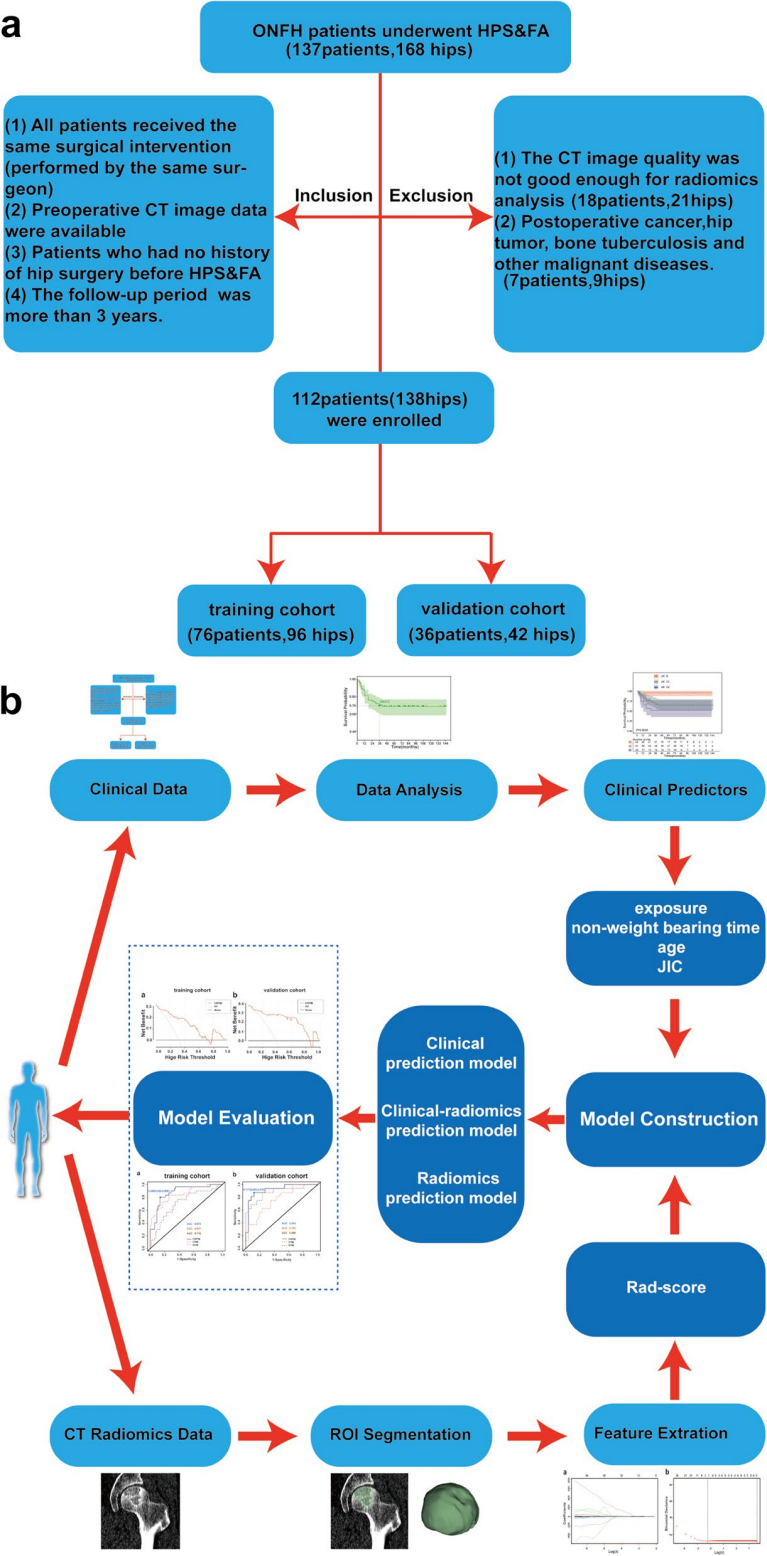
**a** Flowchart of study enrollment, **b** Flowchart of the study

According to literature reports, hip preservation failure has been defined as hip replacement within 3 years after HPS, or Harris score < 90 with progressive collapse of the femoral head on imaging. From January 2009 to December 2019, 137 patients (168 hips) with pathologically confirmed ONFH who underwent HPS&FA at our institution were enrolled. The criteria for inclusion and exclusion were as follows: Inclusion criteria (1) All patients got the same surgical intervention (performed by same surgeon); (2) Preoperative CT image data were available; (3) Patients had no history of hip surgery prior to HPS&FA; (4) The duration of follow-up was greater than three years. Exclusion criteria: (1) Insufficient CT picture quality for radiomics analysis; (2) Postoperative cancer, hip tumor, bone tuberculosis, and other malignant disorders. This study used the hip as a unit and comprised 138hips from 112 patients. Then, the entire dataset was randomly divided into a training cohort (*n* = 96) and a validation cohort (*n* = 42) with a ratio of 7:3 using computer-generated random numbers [15, 16]. The Institutional Review Board and Human Ethics Committee approved this retrospective study and waived the requirement to obtain written informed consent. The case selection process is shown in Fig. 1a, and the flowchart of study is shown in Fig. 1b.

### Clinical data

General data (age, gender, affected side, disease duration, body mass index (BMI), pathogenic factors), preoperative examination indexes (D-dimer, alkaline phosphatase (ALP), white blood cell count (WBC), neutrophil percentage(N), *α*-L-fucosidase (AFU)), ARCO stage, JIC classification, and preoperative Harris score were gathered and recorded. Following HPS&FA, patients were followed up to see if they continued use of glucocorticoids or alcohol, non-weightbearing time, and whether they underwent hip replacement. Additionally, postoperative Harris score and X-ray were assessed every 3 months for the first year and then, every 6 months after that. Ultimately, on June 30, 2022, every piece of data was reviewed.

### CT radiomics data

The Picture Archiving and Communication (PCAS) system was utilized to acquire images. In addition, all patients underwent CT examinations of the hip utilizing the Philips 128-row Brilliance CT and the GE 64-row LightSpeed VCT. Scan parameters include tube voltage 110–150 kV, tube current 220–680 mA, exposure time 240–800 ms, slice thickness 1.0–3.0 mm, slice spacing 1.0–3.0 mm, and reconstruction matrix 512 × 512.

**Fig. 2:**
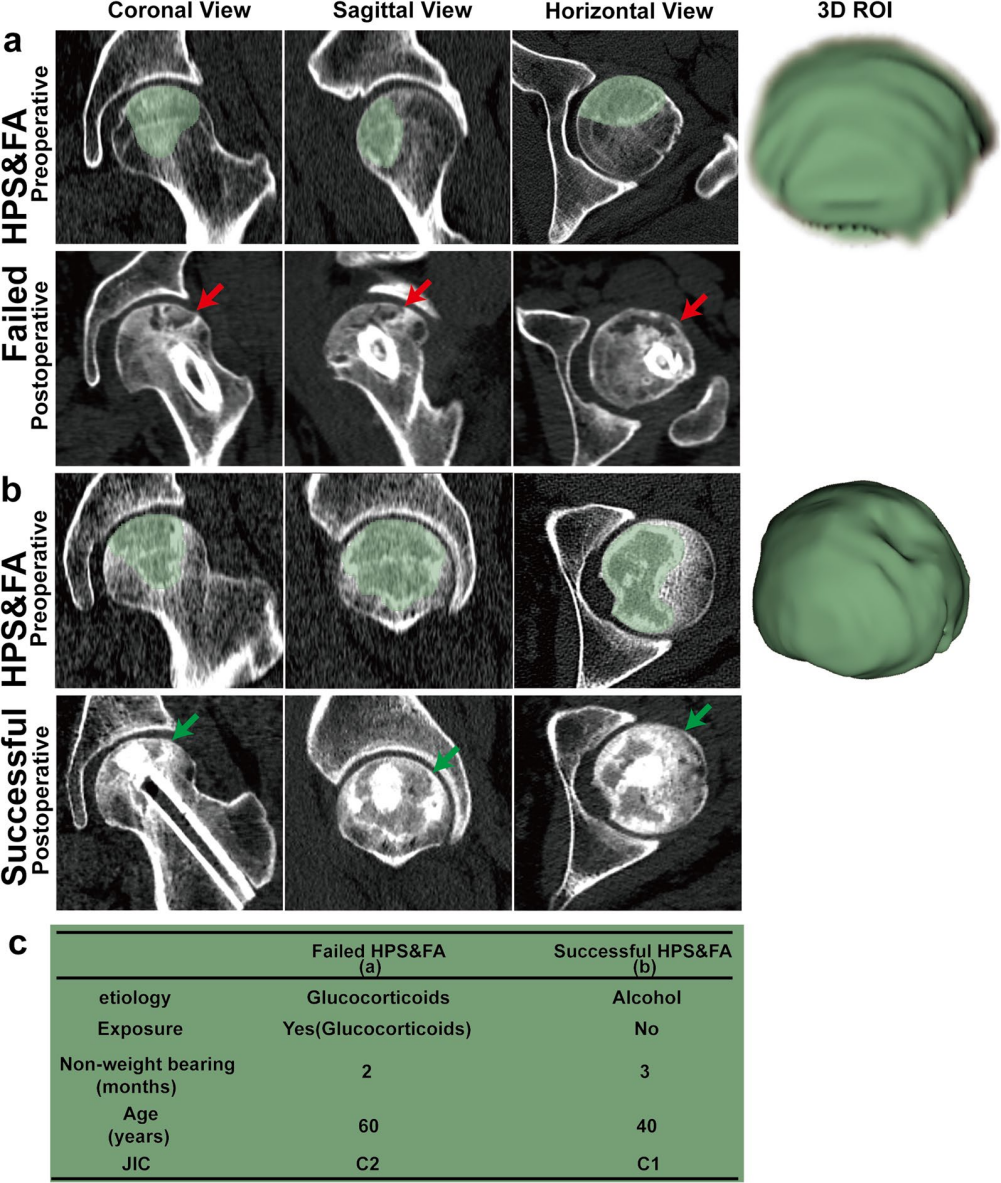
Representative CT images for failed **a** HPS&FA and successful **b** HPS&FA. **c** Comparison of basic information and risk factors. Red arrow: necrosis volume was large and involved the lateral column in (**a**). Green arrow: necrosis volume was relatively small and the lateral column was not accumulated in (**b**)

We then segmented and extracted features from CT images. Bilinear interpolation was used for resampling, with layer thickness and spacing of 1 mm, imported into 3D Slicer (https://www.slicer.org, V5.0.2) as NII files. This study focused on femoral head necrosis, defined as fractures of the trabecular bone, texture disorders, sclerosis zones surrounding low-density areas, and cystic degeneration on CT images (sagittal, coronal and horizontal). Reader 1 (Xin Liu) and Reader 2 (Bin Du) outlined this ROI (Fig. 2) in the bone window of CT images. (Hounsfiled Unit (HU)) value was set at 1500HU, window level at 400U. Pyradiomics plugin automatically extracted 851 imaging features. Radiomics features extracted by two readers were evaluated using ICC. Consistency is deemed satisfactory when ICC > 0.75. Reader 1 segmented 30 CT pictures twice within one month to compute intra-observer ICC. Reader 2 segmented selected images separately to calculate inter-observation ICC. We calculated intra-observer and inter-observer ICC. There was no statistically significant difference between Reader 1 and Reader 2. The intra- and inter-observer ICC exceeded 0.75. Thus, both intra- and inter-observer feature extraction exhibited high repeatability. Eventually, all results were based on measurements made by Reader 1. Then, Z-score normalization was applied to guarantee repeatability.

### Clinical predictors & Rad-score

Both clinical and CT radiomics data were screened. Using univariate and multivariate analysis, clinical predictors were quickly extracted from 16 clinical data. In addition, the LASSO was utilized to determine the optimal radiomics features among CT radiomics features. The optimal radiomics features with nonzero coefficients were ultimately linearly combined to yield a Rad-score for classification analysis.

### Prediction model construction

Clinical data and Rad-score were utilized for modeling by using R statistical software, and then, two prediction models were established, namely CPM and RPM. Subsequently, in order to improve model prediction performance whenever possible, a comprehensive model was developed by integrating clinical predictors with Rad-score as the predictive model for HPS&FA, namely CRPM. At last, using DeLong test, the significance of differences in AUC between models was determined.

### Performance assessment of the models

ROC and AUC were plotted to analyze the diagnostic efficacy of model. Then, to visualize the relationship between the variables in the prediction model, a nomogram based on CRPM for individualized efficacy prediction was constructed. Furthermore, a calibration curve was developed to evaluate the calibration utility of nomogram. To evaluate the medical benefit of nomogram under different risk thresholds, DCA was employed. The model was finally validated using the validation cohort.

### Statistical analysis

For statistical analysis and model development, SPSS 26.0 and R statistical software (version 1.2.5042) were employed. Using SPSS, both univariate and multivariate analyses were conducted. The R software "glmnet" package was used to perform LASSO Using the "survminer" package for proportional hazards model (COX) survival analysis in order to visualize the relationship between variables and determine the cut-off value. ROC and AUC were then plotted using the "pROC" package, and a nomogram was constructed using the "rms" package. We drew calibration and decision curves for the accuracy and clinical utility of prediction models, respectively, using the "rmda" package.

## Results

### Clinical characteristics and CT radiomics features

The study enrolled 138 hips (112 patients). Statistically significant differences between training and validation cohorts were not found in 16 clinical data analyses (Table1). From 16 clinical data, we identified four clinical predictors using univariate and multivariate analysis. Specifically, there were significant differences in age (*P* = 0.020), JIC classification (*P* = 0.019), postoperative continued use of glucocorticoids or alcohol (*P* = 0.001), and postoperative complete non-weightbearing time (*P* = 0.031) between successful and unsuccessful HPS&FA patients (Table 2).

**Table 1:** Comparison of clinical data between training cohort and validation cohort

Variable	Training cohort (*n* = 96)		Validation cohort(*n* = 42)		*P*
	Successful	Failed	Successful	Failed	
*Gender(n,%)*					0.507
Male	51(67.1)	25(32.9)	22(62.9)	13(37.1)	
Female	15(75.0)	5(25.0)	4(57.1)	3(42.9)	
*Affected side(n,%)*					0.307
Left	36(63.2)	21(36.8)	13(61.9)	8(38.1)	
Right	30(76.9)	9(23.1)	13(61.9)	8(38.1)	
*Exposure(n,%)*					0.857
Yes	8(30.8)	18(69.2)	6(50.0)	6(50.0)	
No	58(82.9)	12(17.1)	20(66.7)	10(33.3)	
*Etiology(n,%)*					0.275
Glucocorticoids	31(77.5)	9(22.5)	12(57.1)	9(42.9)	
Alcohol	9(45.0)	11(55.0)	5(100.0)	0(0.0)	
Traumatic	25(73.5)	9(26.5)	7(53.8)	6(46.2)	
Idiopathic	1(50.0)	1(50.0)	2(66.7)	1(33.3)	
*ARCO(n,%)*					0.480
IIA	48(72.7)	18(27.3)	23(69.7)	10(30.3)	
IIB	16(61.5)	10(38.5)	3(37.5)	5(62.5)	
III	2(50.0)	2(50.0)	0(0.0)	1(100.0)	
*JIC classification(n,%)*					0.232
Type B	21(95.5)	1(4.5)	11(84.6)	2(15.4)	
Type C1	33(64.7)	18(35.3)	14(58.3)	10(41.7)	
Type C2	12(52.2)	11(47.8)	1(20.0)	4(80.0)	
Age(years,M ± SD)	37.70 ± 12.31	44.33 ± 11.14	40.42 ± 10.38	43.25 ± 9.21	0.423
Nonbearing(months,M ± SD)	4.26 ± 1.48	3.53 ± 1.28	4.27 ± 1.51	4.69 ± 1.54	0.147
Progress(months,M ± SD)	5.82 ± 7.81	5.67 ± 4.86	5.96 ± 13.60	4.11 ± 5.57	0.740
Harris(score,M ± SD)	70.44 ± 9.11	71.77 ± 10.81	70.50 ± 9.42	66.31 ± 15.02	0.311
BMI(kg/m2,M ± SD)	23.86 ± 2.31	23.97 ± 1.77	22.95 ± 1.57	23.75 ± 2.57	0.104
D-dimer(mg/L, M ± SD)	0.39 ± 0.29	0.71 ± 0.93	0.68 ± 1.60	0.63 ± 0.54	0.284
WBC(× 109/L,M ± SD)	7.14 ± 1.74	6.81 ± 1.26	6.80 ± 0.92	7.10 ± 0.91	0.636
N(%, M ± SD)	62.12 ± 9.83	63.16 ± 10.56	59.46 ± 9.26	58.52 ± 15.97	0.093
ALP(U/L, M ± SD)	94.39 ± 26.46	90.80 ± 24.96	87.65 ± 25.75	100.3 ± 28.46	0.871
AFU(U/L, M ± SD)	17.37 ± 6.05	18.25 ± 8.15	16.36 ± 5.72	20.34 ± 9.12	0.857

**Table 2:** Univariable and multivariable analysis of training cohort

Variable	Univariable analysis			Multivariable analysis		
	HR	95%CI	*P*	HR	95%CI	*P*
Exposure	0.09	0.03–0.26	< 0.001	0.148	0.047–0.462	0.001
P1ace	0.51	0.21–1.29	0.147			
Age	1.05	1.01–1.09	0.011	1.067	1.010–1.126	0.020
Gender	0.68	0.22–2.08	0.486			
Etiology	1.16	0.73–1.85	0.522			
Progress	1.00	0.94–1.06	0.912			
ARCO	1.65	0.78–3.49	0.220			
JIC classification	3.00	1.46–6.16	0.001	2.789	1.181–6.584	0.019
Non-weightbearing	0.68	0.48–0.96	0.017	0.590	0.365–0.953	0.031
Harris	1.01	0.97–1.06	0.561			
BMI	1.02	0.84–1.25	0.801			
DD	3.13	0.96–10.2	0.072			
WBC	0.88	0.66–1.16	0.294			
N	1.01	0.97–1.06	0.649			
ALP	0.99	0.98–1.01	0.524			
AFU	1.02	0.96–1.09	0.601			

**Fig. 3:**
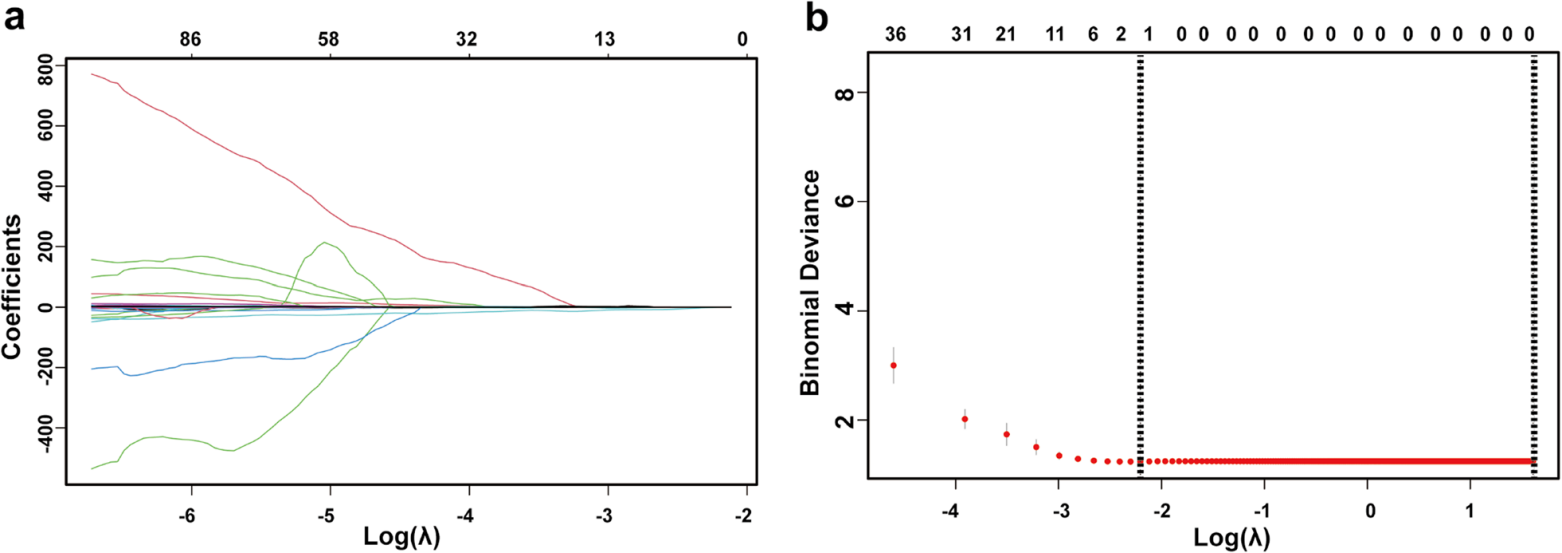
Procession of LASSO. **a** Regression coefficient plot, **b** Cross-validation plot

In this study, LASSO was used to reduce dimension and screen radiomics features, as depicted in Fig. 3. Following are the two radiomics characteristics most closely associated with the endpoint of HPS&FA: wavelet-HLH glcm Idmn and original shape Minor Axis Length. In addition, the formula for the radiomics score depends on the weight coefficients of each feature, as shown below:

Rad-score = 6.889501813–0.009149794*original_shape_MinorAxisLength-5.302489854*wavelet-HLH_glcm_Idmncc.

### Survival analysis of predictors

**Fig. 4:**
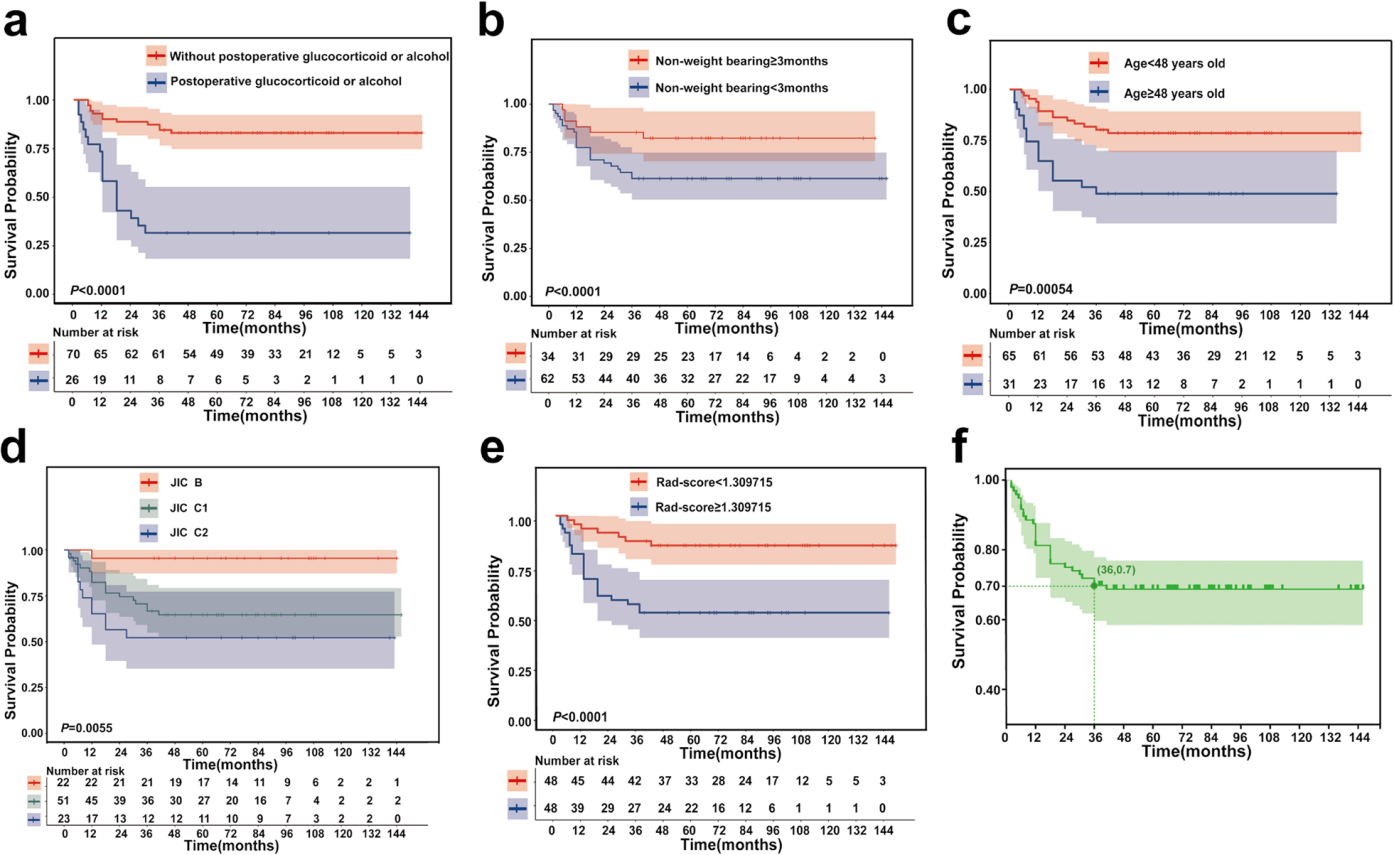
Kaplan–Meier analyses of the 5 selected features for patients in the training cohort. **a** Exposure **b** non-weightbearing **c** age **d** JICclassification **e** Rad-score **f** The survival rate after HPS&FA is basically stable at 36 months

In Fig. 4, Kaplan–Meier survival curves showed that mass failures were concentrated in the first 24 months. After 36 months, the femoral head survival rate was stable. HPS&FA success rate was 69.57%, and 46 out of 138 hips failed. There were 26 failures within 12 months, 10 failures between 12 and 24 months, 7 failures between 24 and 36 months, and only three failures after 36 months. A 36-month time endpoint was used for evaluating HPS&FA effectiveness. Moreover, Kaplan–Meier analysis found HPS&FA failures were significantly increased by age greater than 48 years, JIC type C, continued use of glucocorticoids or alcohol postoperatively, complete non-weightbearing time less than 3 months, and Rad-scores greater than 1.309715.

### Model construction and comparison

**Fig. 5:**
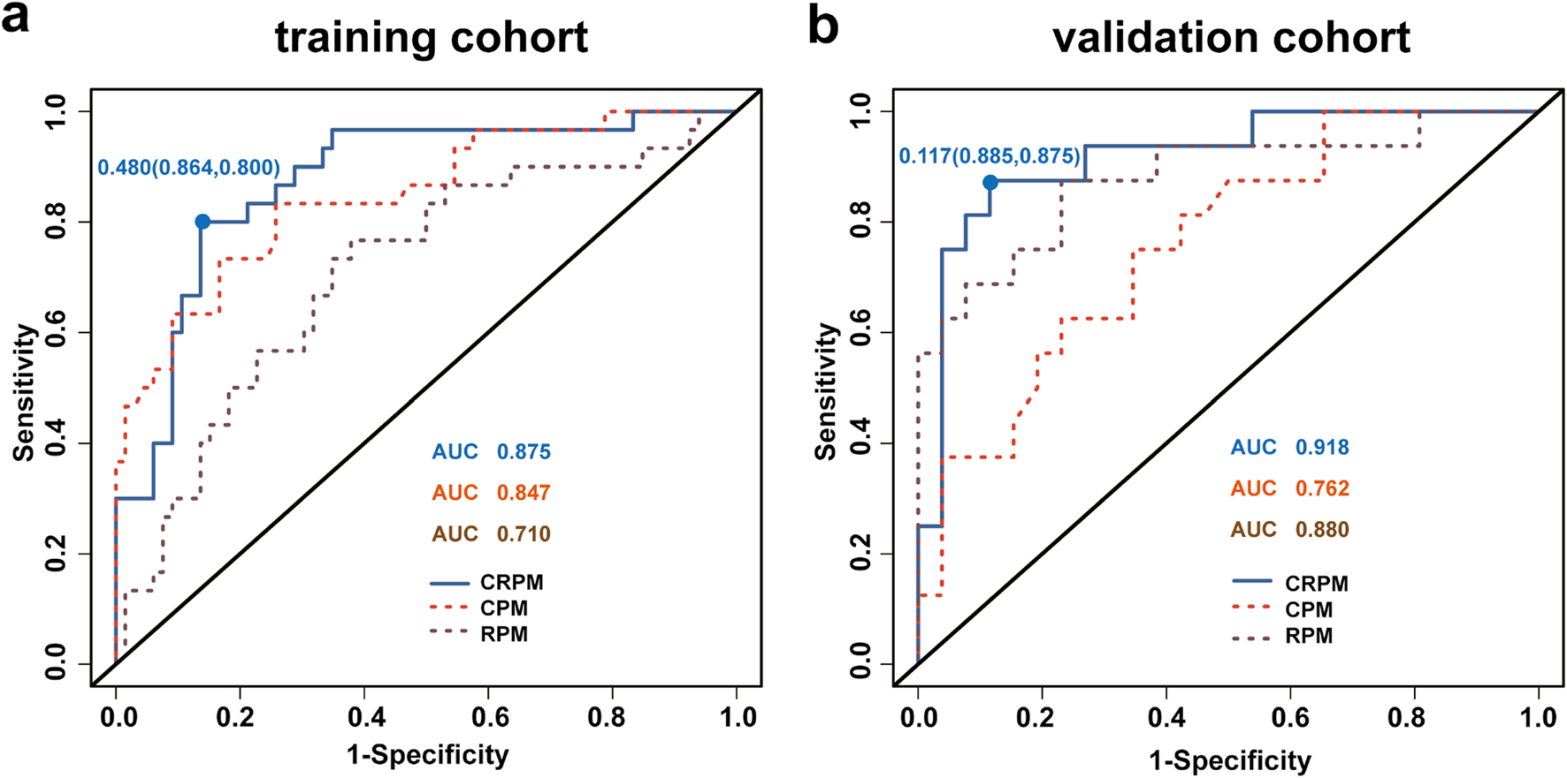
ROC for different models in the training (**a**) and validation (**b**) cohorts

ROC curves of the prediction models in training and validation cohorts were plotted to identify prediction performance (Fig. 5). AUC of CRPM was greater than that of CPM and RPM in both cohorts. In training cohort, AUC of CRPM was 0.875, the predictive sensitivity was 0.800 and the specificity was 0.864 at the best cut-off point of 0.480. AUC of CRPM in the validation cohort was 0.918, the predictive sensitivity was 0.875 and the specificity was 0.885 at the best cut-off point of 0.117. (Table 3).

**Table 3:** Performance evaluation of training and validation cohorts, including specificity sensitivity and 95% confidence interval

Model Type	Training cohort(*n* = 96)			Validation cohort(*n* = 42)		
	sensitivity	specificity	AUC(95%CI)	sensitivity	specificity	AUC(95%CI)
RPM	0.767	0.621	0.710(0.596–0.824)	0.875	0.769	0.880(0.763–0.996)
CPM	0.833	0.742	0.847(0.760–0.933)	0.750	0.654	0.762(0.615–0.909)
CRPM	0.800	0.864	0.875(0.799–0.950)	0.875	0.885	0.918(0.829–1.000)

DeLong test revealed a significant difference between CRPM and RPM in training cohort (*P* = 0.004675), but none between CRPM and CPM (*P* = 0.2224). There was a significant difference between CRPM and CPM in validation cohort (*P* = 0.03674), but no significant difference between CRPM and RPM (*P* = 0.3263). According to the combined results of DeLong test, ROC, and AUC, CRPM model that incorporated clinical predictors and Rad-score had the superior predictive ability.

### Assessment and validation of CRPM

**Fig. 6:**
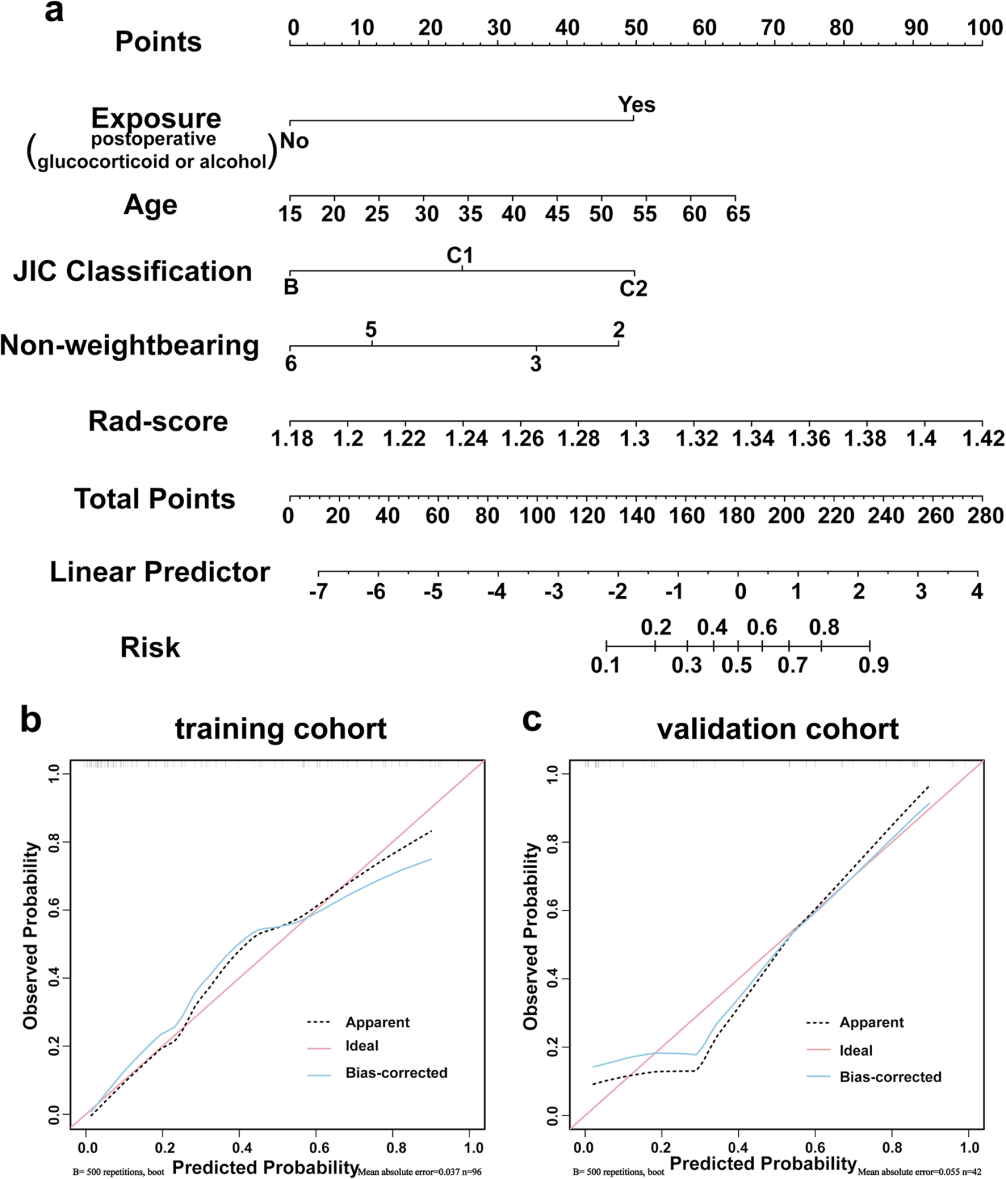
**a** Nomogram of the CRPM for predicting the efficacy of HPS&FA. **b**, **c** Calibration curves of CRPM in the training (**b**) and validation (**c**) cohorts

CRPM was visualized as a nomogram (Fig. 6a) to better evaluate the predictors. The nomogram demonstrated that the predicted scores of patients in the training cohort were consistent with clinical reality. Younger age, JIC type B of the femoral head, avoiding continued use of glucocorticoids or alcohol after surgery, complete non-weightbearing time close to 6 months after surgery, and a smaller Rad-score significantly increase the success rate of HPS&FA. The calibration curves of the CRPM training and validation cohorts revealed similar significant agreement between estimation and practical observation (Fig. 6b, c).

### Clinical use of CRPM

**Fig. 7:**
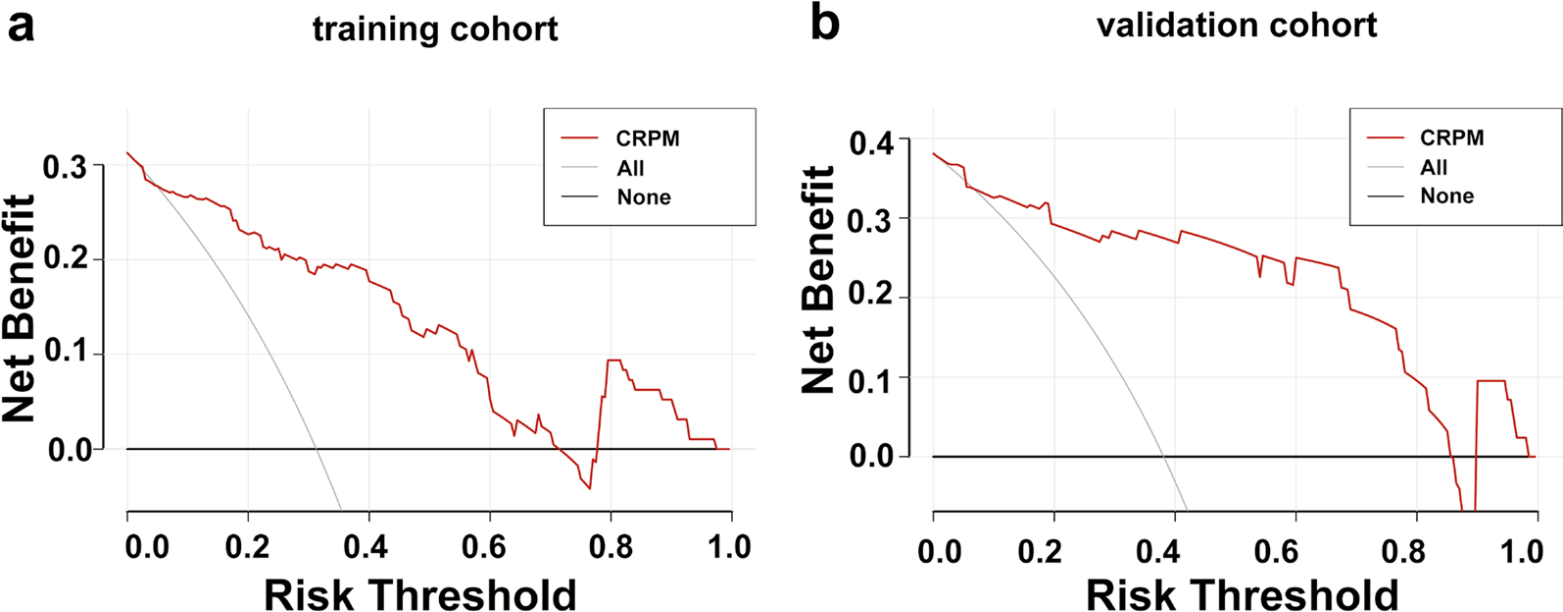
DCA of CRPM in training (**a**) and validation (**b**) cohorts

Figure 7 depicts the DCA of CRPM. If the patient's risk threshold is greater than 5%, CRPM could add more benefit than no treatment option or an all-patient treatment option in most situations. DCA in validation cohort was slightly unsatisfactory, but the trend was similar to that in training cohort, which could bring more net benefits to patients within a wide range of risk thresholds.

## Discussion

The CRPM demonstrated superior performance compared to both the RPM and CPM. Using CT radiomics, RPM can provide detailed information on the necrotic area of the femoral head. Instead, CPM evaluates the patient's physical condition, lifestyle, and laboratory tests. As a result of combining the benefits of both models and improving prediction accuracy, the CRPM is an improved model for predicting early postoperative efficacy. In contrast to previous research, we incorporated as many clinical markers as possible. Furthermore, to quantitatively differentiate patients, we evaluated the critical value of predictors in order to significantly improve prediction. Simultaneously, the predictive parameters were incorporated into the nomogram for visualization, facilitating clinical application. HPS&FA nomogram not only predicts effectiveness but also guides perioperative care. With better perioperative supervision, surgeons can choose patients for HPS&FA based on preoperative predictions (age, JIC classification, CT imaging). Further, postoperative predictors can be utilized to direct postoperative rehabilitation, such as glucocorticoids or alcohol usage.

HPS&FA efficacy was influenced by four clinical predictors included in CRPM. First, glucocorticoids and alcohol use after surgery have been associated with hip preservation failure (*P* < 0.0001). In clinical practice, glucocorticoids-associated osteonecrosis of the femoral head (GA-ONFH) is most prevalent [17, 18]. Modern studies have demonstrated that glucocorticoids and alcohol can lead to decreased osteogenic capacity, sparse bone trabeculae, and decreased bone density [19]. In addition, it can cause microcirculation disturbances in the femoral head, leading to local metabolic abnormalities that delay or fail bone reconstruction [20, 21]. Secondly, in order to provide a relatively stable environment for bone regeneration, the affected side must refrain from bearing weight for a period of time following surgery (*P* < 0.0001) [22, 23]. Crawling-replacement of bone trabeculae and angiogenesis occur during this period. As bone repair of the femoral head requires 3–6 months, premature weightbearing will put excessive pressure on the femoral head, causing the bone repair process to fail. After three months of non-weightbearing, partial weightbearing is recommended. In our survival analysis, three months was the cut-off value. It is consistent with clinical experience that a shorter period of non-weightbearing time increases failure risk. Third, age is an objective factor (*P* < 0.00054). As a person ages, osteoclast activity gradually exceeds osteogenic activity due to an imbalance in bone metabolism [24, 25]. As a result, bone strength decreases and bone fragility increases. Age also contributes to slow or failed postoperative bone repair. The last clinical factor is JIC classification (*P* < 0.0055), JIC classification is a classic clinical stage system for ONFH, which is characterized by three columns of necrosis according to the extent of necrosis [26]. Nomogram results indicate that patients with lateral column necrosis generally have a poor prognosis after HPS&FA, which is in line with clinical studies [27]. Rad-score (*P* < 0.0001) derives from in-depth exploration of CT radiomics. A necrotic area's location and volume influence its risk. Literatures indicate that HPS&FA efficacy depends on the area and size of the necrotic area preoperatively. A large necrotic area as well as a necrotic area close to the cartilage in the weightbearing area increases the risk of hip preservation surgery failure [28]. Numerous studies have sought to analyze and quantify the necrotic area's morphology, but the results have not fully reflected the actual morphology of the necrotic area [29–31]. There was no solution to this problem until radiomics. The first three clinical risk factors can more accurately predict postoperative efficacy among the four. JIC classification is the fourth risk factor. Although JIC classifications are objectively determined by doctors based on imaging examinations, they are distorted by numerous variables (imaging data differences, individual differences among doctors) and cannot fully reflect the results of imaging examinations. We would not have chosen HPS&FA for patients with JIC simple medial type, which would have diminished JIC's predictive strength, making it appear that it was not the best predictor. Rad-score, generated from Radiomics, was virtually identical to P values of postoperative exposure and weight-free time, indicating that Rad-score could be combined with clinical risk factors to create CRPM. Because the Rad-score is derived from numerous imaging features, it reflects imaging findings more accurately than JIC classification. Though both JIC and Rad-score are derived from imaging data, but given the widespread popularity of JIC, we take both of them. In this paragraph, we explain how the CRPM we developed can accurately predict postoperative efficacy, providing a solution to the problem of predicting HPS&FA postoperative efficacy. To inform preoperative patient screening and personalized perioperative interventions, we will conduct a comprehensive analysis of CRPM predictive factors.

CRPM can predict HPS efficacy as well as guide patient screening and personalized perioperative interventions to improve HPS&FA. According to survival analysis and the nomogram, JIC classification, age, and Rad-score are all positively correlated with hip preservation failure. Consequently, those with a high JIC classification, older than 48 years, and a Rad-score 1.309715 should be cautiously included. Once HPS&FA has been performed, the use of glucocorticoids or alcohol and non-weightbearing time influence HPS&FA's effectiveness. The nomogram suggests avoiding glucocorticoids or alcohol and extending non-weightbearing time can increase HPS&FA success. If you need to continue using glucocorticoids or alcohol after surgery, we strongly recommend extending non-weightbearing time to improve HPS&FA success. Through the specific analysis of preoperative and postoperative risk factors and interventions, we can better screen out suitable patients. Furthermore, we can improve HPS&FA success rates with perioperative advance intervention.

There are some limitations to our study: (1) It was a single-center, regressive study, with a relatively small number of included cases, and possible errors. Further verification of multicenter, large-sample, and prospective studies is needed; (2) Failure to quantify the same surgical intervention between different surgeons to increase sample size, which might be addressed by standardized surgical technology assessment tools. (3) Since this study was not prospective, patients had been screened prior to surgery, which may have eliminated some risk factors.

## Conclusion

In conclusion, this study developed CRPM, a clinical predictor based on radiomics that could predict the effectiveness of HPS&FA, provide patients screening and personalized perioperative intervention to improve HPS&FA success. In addition, CRPM is clinically practicable and effective and is easy to be popularized and applied after visual nomogram display.

## Data Availability

The datasets used and/or analyzed during the current study are available from the corresponding author on reasonable request.

## Abbreviations

ONFH: Osteonecrosis of femoral head
HPS: Hip preservation surgery
HPS&FA: Hip preservation surgery with fibula allograft
CT: Computed tomography
CPM: Clinical prediction model
RPM: Radiomics prediction model
CRPM: Clinical-radiomics prediction model
LASSO: Least absolute shrinkage and selection operator
ROC: Receiver operating characteristic curve
AUC: Area under the curve
DCA: Decision curve analysis
JIC: Japanese investigation committee
BMI: Body mass index
ALP: Alkaline phosphatase
WBC: White blood cell count
N: Neutrophil percentage
AFU: Fucosidase
ROI: Region of interest
ICC: Intraclass correlation coefficients
